# A novel decision model to predict the impact of weight management interventions: The Core Obesity Model

**DOI:** 10.1002/osp4.495

**Published:** 2021-03-09

**Authors:** Sandra Lopes, Henrik H. Meincke, Mark Lamotte, Anamaria‐Vera Olivieri, Michael E. J. Lean

**Affiliations:** ^1^ Novo Nordisk A/S Søborg Denmark; ^2^ IQVIA Zaventem Belgium; ^3^ IQVIA Basel Switzerland; ^4^ Human Nutrition School of Medicine, Dentistry and Nursing Royal Infirmary University of Glasgow Glasgow UK

**Keywords:** cost‐effectiveness, health economics, obesity therapy

## Abstract

**Aims:**

Models are needed to quantify the economic implications of obesity in relation to health outcomes and health‐related quality of life. This report presents the structure of the Core Obesity Model (COM) and compare its predictions with the UK clinical practice data.

**Materials and methods:**

The COM is a Markov, closed‐cohort model, which expands on earlier obesity models by including prediabetes as a risk factor for type 2 diabetes (T2D), and sleep apnea and cancer as health outcomes. Selected outcomes predicted by the COM were compared with observed event rates from the Clinical Practice Research Datalink‐Hospital Episode Statistics (CPRD‐HES) study. The importance of baseline prediabetes prevalence, a factor not taken into account in previous economic models of obesity, was tested in a scenario analysis using data from the 2011 Health Survey of England.

**Results:**

Cardiovascular (CV) event rates predicted by the COM were well matched with those in the CPRD‐HES study (7.8–8.5 per 1000 patient‐years across BMI groups) in both base case and scenario analyses (8.0–9.4 and 8.6–9.9, respectively). Rates of T2D were underpredicted in the base case (1.0–7.6 vs. 2.1–22.7) but increased to match those observed in CPRD‐HES for some BMI groups when a prospectively collected prediabetes prevalence was used (2.7–13.1). Mortality rates in the CPRD‐HES were consistently higher than the COM predictions, especially in higher BMI groups.

**Conclusions:**

The COM predicts the occurrence of CV events and T2D with a good degree of accuracy, particularly when prediabetes is included in the model, indicating the importance of considering this risk factor in economic models of obesity.

## 1 INTRODUCTION

The high prevalence and chronic nature of obesity are compounded by the large number of related complications.^1^ There is extensive evidence of a link between body mass index (BMI) and type 2 diabetes (T2D), as well as cardiovascular disease (CVD), including both chronic complications, such as hypertension and coronary heart disease, and acute events such as myocardial infarction (MI) and stroke.^1^ ^,^ ^2^ Furthermore, obesity is associated with other complications across multiple organ systems, including sleep apnea^3^ and osteoarthritis, and is also implicated in the development of some types of cancer.^4^ These complications incur a substantial proportion of obesity‐related healthcare costs.^5^, ^6^, ^7^

Health economic models of obesity are used to assess the cost‐effectiveness of weight management interventions, driving healthcare decision‐making and allocation of resources. To do this, such models estimate the risk of BMI‐related complications, the impact on health‐related quality of life (HRQoL), and the associated economic costs.^8^ Obesity models set in the UK healthcare system, assessing the long‐term impact of T2D and CVD, and also incorporating mortality, have previously been developed, principally for use in economic predictions. These models have been used to assess the cost‐utility of orlistat^9^ and compare the cost‐effectiveness of orlistat, sibutramine, and rimonabant,^8^ and to assess the cost‐effectiveness of the LighterLife weight management program^10^ and the Weight Action Program^11^ However, these previous models can be refined and improved upon; given the multifactorial nature of overweight and obesity and the range of associated complications, the incorporation of additional comorbidities and risk factors offers the potential to improve the accuracy of predictions. Furthermore, models must be fit for purpose and interpretable by key stakeholders. This need for transparency and accuracy in model development has informed published best practice guidelines by the International Society for Pharmacoeconomics and Outcomes Research (ISPOR) and the Society for Medical Decision Making (SMDM).^12^ ^,^ ^13^

This report presents the development and structure of the novel Core Obesity Model (COM; version 8.0). The COM was designed to encompass a broader range of complications than previous models and includes the impact of sleep apnea, knee replacement as a result of osteoarthritis, postmenopausal breast cancer, postmenopausal endometrial cancer, and colorectal cancer. The model also incorporates the impact of prediabetes, which is known to be associated with increased all‐cause mortality, as well as a higher risk of T2D and CVD.^14^

Furthermore, the results of an analysis comparing model predictions with observed rates of obesity‐related complications in the UK clinical practice data are presented, to demonstrate the functionality of the COM and assess the impact of baseline glycemic status on its predictions while highlighting areas for further refinement of the model.

## 2 MATERIALS AND METHODS

### 2.1 Core Obesity Model overview

#### 2.1.1 Model structure

**FIGURE 1:**
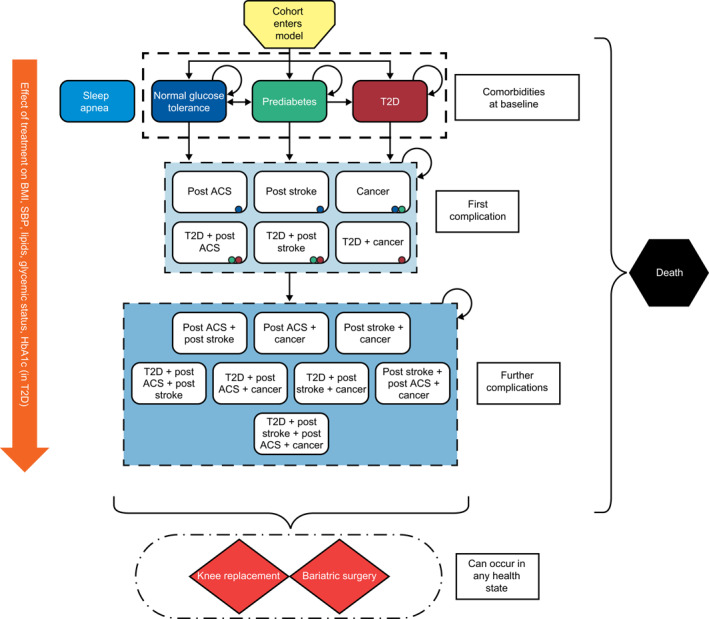
Structure of the Core Obesity Model. This model schematic was previously published as part of a manuscript describing the validation of the Core Obesity Model30 and is reproduced here in accordance with the Creative Commons Attribution‐Non‐Commercial 4.0 International License (http://creativecommons.org/licenses/by‐nc/4.0/) and with the permission of the copyright holders (authors). ACS, acute coronary syndrome; BMI, body mass index; HbA1c, glycated hemoglobin; SBP, systolic blood pressure; T2D, type 2 diabetes

The COM is a Markov, closed‐cohort model. In a Markov model, the disease being studied is divided into distinct and mutually exclusive states (health states) and transition probabilities are assigned to represent patients moving between these states over discrete time periods called “Markov cycles”. By applying these transitions in the model and attaching estimates of resource use and health consequences to the states, followed by running the model over a large number of cycles, it is possible to estimate the longitudinal costs and outcomes associated with the disease. The health states are chosen to represent clinically and economically important events in the disease process. The states are mutually exclusive: a patient can only be in one state at a time and cannot transition to a less severe health state.^15^ The COM comprises 18 single or combined obesity‐related health states (Figure 1), intended to reflect the disease course and impact of effective weight management interventions for individuals who are currently living with BMI above 25 kg/m^2^.^16^ The model was originally developed in Microsoft Excel 2013. The structure incorporates key findings from previous obesity models and has been refined by reviewing the relevant literature and incorporating expert clinical feedback to establish face validity.^13^ A cohort state‐transition model was chosen to avoid the need for extensive code and computational intensiveness associated with microsimulation models^17^ while maximizing transparency and user‐friendliness.

Patients enter the model in a defined baseline health state (i.e., no comorbidity, prediabetes, or T2D) and at each model cycle can either remain in the same state or transition to another state. The time horizon of the model is 40 years, chosen to represent a lifetime time horizon for most of the cohort commencing weight management and entering the model. Transition probabilities are based on risk equations or risk tables for each obesity‐related complication from published landmark epidemiological studies and linking surrogate endpoints, such as BMI, systolic blood pressure (SBP), lipids, glycemic status, and for those with T2D, glycated hemoglobin (HbA1c) to “hard” clinical outcomes, such as CVD and diabetes. These surrogate endpoints are risk factors for obesity‐related complications and typical endpoints in relevant clinical trials.

The effect of weight management interventions on transition probabilities between health states is incorporated via the observed effect that these interventions have on BMI and cardiometabolic risk factors (Table 1). Third‐party payer, patient and societal costs, and HRQoL data associated with interventions and health states are also incorporated into the model.

**TABLE 1:** Definition of treatment effects on physiological parameters included in the Core Obesity Model

Surrogate outcomes	Treatment effect included in the model
BMI, kg/m^2^	BMI percentage change from baseline (note: percentage weight change in kg is equal to percentage BMI change)
SBP, mmHg	SBP absolute change from baseline
HDL cholesterol, mg/dL	HDL cholesterol absolute change from baseline
Total cholesterol, mg/dL	Total cholesterol absolute change from baseline
HbA1c, %, in diabetes	HbA1c percentage‐point change from baseline

Abbreviations: BMI, body mass index; HbA1c, glycated hemoglobin; HDL, high‐density lipoprotein; SBP, systolic blood pressure.

#### 2.1.2 Baseline characteristics

The baseline characteristics of a hypothetical patient cohort (e.g., age, sex, and cardio‐metabolic risk factors: BMI, SBP, lipids, and HbA1c level [for those with T2D]) are defined at model entry (Table 2), and are classified as either static (do not change over time) or dynamic (change over time).

**TABLE 2:** Summary of cardio‐metabolic risk factors included in the Core Obesity Model

	Unit of measure	Parameter nature	Description
Age	years	Dynamic	Defined at baseline and increasing by 1 unit each year spent alive in the cohort
BMI	kg/m^2^	Dynamic	Defined at baseline; changes as a result of treatment; when treatment is stopped weight is regained after a defined period (catch‐up period) and afterwards has a natural progression (increase) until a predefined age
Height	cm	Static	Defined at baseline, does not change
SBP	mmHg	Dynamic	Defined at baseline, changes as a result of treatment (if decreased due to treatment, catch up after treatment stop is assumed)
Total cholesterol	mg/dl	Dynamic	Defined at baseline, changes as a result of treatment (if decreased due to treatment, catch up after treatment stop is assumed)
HDL cholesterol	mg/dl	Dynamic	Defined at baseline, changes as a result of treatment (if increased due to treatment, catch up after treatment stop is assumed)
HbA1c in cohort with type 2 diabetes	%	Dynamic	Defined at baseline, changes as a result of treatment. When the entire cohort has diabetes, following treatment stop and catch‐up period, HbA1c increases over time based on natural progression in diabetes population
Type 2 diabetes duration in cohort with type 2 diabetes	years	Dynamic	Defined at baseline and increasing by 1 each year spent alive in the cohort
Triglyceride level	‐	Static	Defined at baseline, does not change
Proportion with triglyceride level ≥150 mg/dl	%	Static	Defined at baseline, does not change
Proportion smokers	%	Static	Defined at baseline, does not change
Proportion women	%	Static	Defined at baseline, changes with mortality
Proportion Mexican Americans (for US cohort only)	%	Static	Defined at baseline, does not change
Proportion receiving lipid‐lowering drugs	%	Static	Defined at baseline, does not change
Proportion receiving antihypertensive medication	%	Dynamic	Defined at baseline, may change as a result of treatment (if decreased owing to treatment, catch up after treatment stop is assumed)
Age at menopause	years	Static	Defined at baseline, does not change

Abbreviations: BMI, body mass index; HbA1c, glycated hemoglobin; HDL, high‐density lipoprotein; SBP, systolic blood pressure.

#### 2.1.3 Health states in the model

The obesity‐related complications included in the model health states are T2D, CVD (consisting of acute coronary syndrome [ACS] and stroke), cancer (postmenopausal endometrial, postmenopausal breast, and colorectal), and death. Additionally, in any given health state, patients can undergo knee replacement surgery because of debilitating osteoarthritis, or undergo bariatric surgery. Sleep apnea was applied at baseline and throughout the time horizon to a proportion of the cohort, and not considered a separate health state, because it can co‐exist with other complications and its onset is not thought to have an impact on mortality or other transition probabilities (Figure 1).^18^

Complications were selected for inclusion in the model because: (1) there is either strong or moderate evidence for their association with obesity (Table 3), based on a comprehensive report from the World Health Organization,^19^ and also referenced in subsequent reports on the burden of obesity‐related conditions;^20^ ^,^ ^21^ (2) they have a considerable impact on HRQoL, life expectancy and/or healthcare resources and costs; and (3) they are known to be affected by weight management interventions.^19^

**TABLE 3:** Summary of obesity‐related complications included in the Core Obesity Model

Complications with strong evidence of association with obesity	Complications with moderate evidence of association with obesity
Type 2 diabetes	Knee replacement
Acute coronary syndrome (including myocardial infarction)	Colorectal cancer
Stroke (including transient ischemic attack)	Postmenopausal endometrial cancer
Sleep apnea	Postmenopausal breast cancer

#### 2.1.4 Transition probabilities and risk equations

Risk equations for transition probabilities were selected using epidemiological studies identified in a systematic literature review (unpublished) conducted by the School of Health and Related Research (Sheffield, United Kingdom) and supplemented with a pragmatic search.

#### 2.1.5 Type 2 diabetes

**FIGURE 2:**
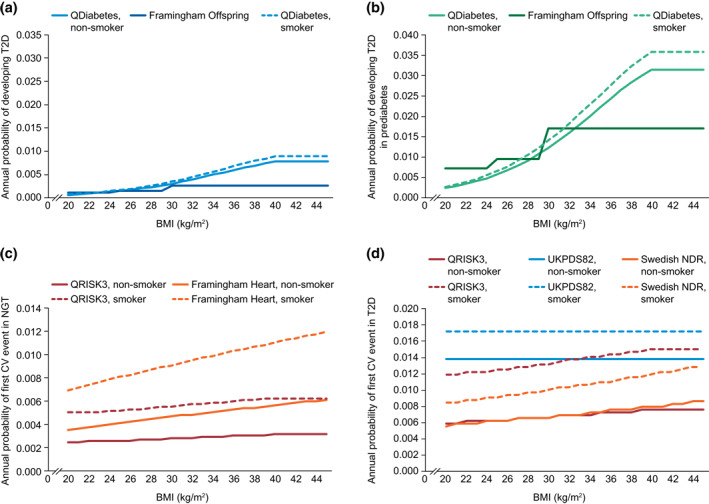
Risk prediction estimates used in the Core Obesity Model. BMI, body mass index; CV, cardiovascular; NDR, National Diabetes Register; NGT, normal glucose tolerance; T2D, type 2 diabetes; UKPDS, UK Prospective Diabetes Study

The two alternative risk equations for the development of T2D in individuals with either no comorbidity or prediabetes were sourced from the QDiabetes study and the Framingham Offspring Study (Figure 2A,B, respectively);^22^ ^,^ ^23^ consequently, risk equations developed in both UK and US populations were used in the model. The QDiabetes‐2018 algorithm predicts the 10‐year risk of T2D in patients aged 25–84 years as a function of BMI and other associated risk factors; the model has been validated externally^22^ and is recommended by the National Institute for Health and Care Excellence (NICE) for T2D risk identification in the United Kingdom.^24^ Compared with the Framingham Offspring Study, the QDiabetes study included a longer prediction range (10 vs. 8 years), wider age range (25–84 years vs. 25–64 years) and higher maximum BMI (40 vs. 30 kg/m^2^).^22^ ^,^ ^23^ To reflect the higher risk of T2D in individuals with prediabetes the HbA1c parameter was set to equal 42 mmol/mol (equivalent to 6%).

#### 2.1.6 Cardiovascular disease and other obesity‐related complications

In the COM, CVD is defined as ACS (includes MI and unstable angina) or stroke (includes transient ischemic attack [TIA]). Different equations can be used to estimate the risk of CVD as either a first‐time or recurrent event. Furthermore, because T2D was included as a risk factor for CVD in all risk‐prediction models, it is possible to differentiate between CVD risk in patients with T2D and risk in those with normal glucose tolerance.

The risk of CVD as a first‐time event may be predicted in the model via four studies. For individuals with normal glucose tolerance, risk of CVD as a first‐time event may be predicted using the QRISK3 study or the Framingham Heart Study (Figure 2C).^25^ ^,^ ^26^ For cohorts from Europe, the estimate from the QRISK3 study is preferred and for those from the United States, the Framingham Heart Study is preferred. Neither of these studies quantifies the risk of CVD in individuals with prediabetes. Therefore, the risk of CVD as a first‐time event was assumed to be the same for individuals with normal glucose tolerance and for those with prediabetes. For individuals with T2D, the QRISK3 study, the United Kingdom Prospective Diabetes Study (UKPDS) outcomes model 2 or the Swedish National Diabetes Registry (Figure 2D) may be used.^27^ ^,^ ^28^

The risk for CVD as a recurrent event was based on estimates from the Framingham Recurring Coronary Heart Disease Study for individuals with normal glucose tolerance and for those with T2D; the UKPDS can be used as an alternative for individuals with T2D.^28^ ^,^ ^29^ To reflect the increased risk of recurrent cardiovascular (CV) events in individuals with impaired glucose tolerance and a history of CV events,^14^ the risk of CVD as a recurrent event for individuals with prediabetes was assumed to be the same as for those with T2D.

Risk estimates for other events and transitions to other health states are summarized in Table 4. These risk equations were selected following identification of relevant studies in the systematic review. Appropriate studies for inclusion were those that focused on relevant populations; were relevant to the countries or regions of interest; reported on the association between BMI, other risk factors relevant to the model, where available, and the outcomes of interest; and were judged to be of high quality. High‐quality studies were considered to be those with appropriate design and modeling, and use of large patient populations to develop and validate risk equations.

**TABLE 4:** Summary of sources used to derive risk estimates for health state transitions in the Core Obesity Model

Complication	Risk estimate source(s)
Sleep apnea	Young et al. 2002^18^
Knee replacement	Wendelboe et al. 2003^30^
Colorectal cancer	Adams et al. 2007^31^
	Schlesinger et al. 2015^32^
Postmenopausal endometrial cancer	Renehan et al. 2008^33^
	Yang et al. 2012^34^
Postmenopausal breast cancer	Ahn et al. 2007^35^
	Renehan et al. 2008^33^

#### 2.1.7 Mortality

General population mortality (defined as age‐ and sex‐specific all‐cause mortality) was included in the model based on country‐specific life tables. Changes to the probability of mortality associated with MI, unstable angina, stroke, knee replacement, and certain cancers were made via adjustments to the general population mortality applied in the COM (Table 5).

**TABLE 5:** Mortality probabilities associated with events and health states in the Core Obesity Model

Model parameter	Estimate mortality probability applied in year of onset		Estimate source(s)	Estimate applied in years post onset^a^	Estimate source(s)
	Female	Male			
Myocardial infarction	30.00%	32.00%	BHF^36^	RR: 1.30	Johansson et al.^37^
Unstable angina	30.00%	32.00%	BHF^36^	RR: 1.30	Johansson et al.^37^
Stroke	24.70%	17.10%	BHF^36^	RR: 2.00	Brammas et al.^38^
Knee replacement	0.30%		Singh et al.^39^ and CRUK^40^	NA	NA
**Cancer:**				Probability: 4.31%	CRUK^40^
Colon	30.11%		CRUK^40^		
Postmenopausal endometrial	10.54%		CRUK^40^		
Postmenopausal breast	4.08%		CRUK^40^		

Abbreviations: BHF, British Heart Foundation; CRUK, Cancer Research UK; NA, not applicable; RR, relative risk.

^a^ Relative risks are applied to the age and sex‐specific annual probabilities of mortality.

### 2.2 CPRD‐HES study and Core Obesity Model comparative analysis

A recently published external validation of the COM showed that it reliably predicts the occurrence of obesity‐related complications.^41^ The aim of this analysis was to assess how baseline glycemic status impacts model predictions using event rates sourced from a large analysis of merged patient data from the Clinical Practice Research Datalink (CPRD), Hospital Episode Statistics (HES), and the Office for National Statistics examining associations between BMI and obesity‐related complications in a cohort of more than 2.9 million individuals followed up for a median of 11.4 years.^42^

#### 2.2.1 Baseline data and model parameters

Individuals in the CPRD‐HES study were stratified into five groups based on conventional BMI cut‐offs, with normal weight (BMI 18.5–24.9 kg/m^2^) as the reference group. The baseline demographic and disease characteristics of these groups (Table 6) were used to populate the model.

**TABLE 6:** Baseline characteristics of the CPRD‐HES BMI groups

BMI group	Normal 18.5–24.9 kg/m^2^	25.0–29.9 kg/m^2^	30–34.9 kg/m^2^	35–39.9 kg/m^2^	40–44.9 kg/m^2^
*N*	1,099,106	1,074,953	507,425	176,237	67,231
Mean age, years (SD)	48.5 (19.2)	53.1 (16.9)	52.1 (15.9)	49.3 (15.4)	47.4 (14.6)
Mean BMI, kg/m^2^ (SD)	22.5 (1.7)	27.3 (1.4)	32.1 (1.4)	37.0 (1.4)	42.3 (1.5)
Mean height, m (SD)	1.68 (0.09)	1.69 (0.10)	1.68 (0.10)	1.66 (0.10)	1.65 (0.10)
Smoking, % ever smoked	49.3	50.2	50.6	48.8	47.3
Sex, % women	64.6	49.7	53.1	62.9	70.9
Individuals on antihypertension medication, %	14.7	22.3	26.1	26.7	28.5
Individuals on lipid‐lowering medication, %	8.3	13.9	15.7	14.6	14.9
Mean SBP, mmHg (SD)	128.2 (71.4)	135.4 (73.8)	138.3 (54.2)	139.4 (56.7)	140.0 (57.7)
Mean total cholesterol, mg/dl (SD)	203.7 (41.5)	207.8 (42.0)	208.0 (42.2)	206.2 (41.7)	201.2 (40.9)
Mean HDL, mg/dl (SD)	61.0 (18.1)	53.9 (16.5)	50.4 (15.3)	48.8 (14.4)	47.5 (14.2)
Mean HbA1c, % (SD)	7.5 (1.6)	7.6 (1.5)	7.7 (1.5)	7.8 (1.5)	7.8 (1.6)
Mean triglycerides, mg/dl (SD)	118.9 (69.0)	152.2 (87.9)	175.0 (98.1)	180.1 (99.4)	177.0 (94.6)
Individuals with triglyceride levels ≥150 mg/dl, %	22.4	40.0	51.7	54.3	53.8
Individuals without pre‐T2D or T2D^a^ ^,^ ^b^, %	95.3	90.2	85.5	82.5	78.8
Individuals with pre‐T2D with laboratory values, %	2.0	4.1	5.9	6.9	6.7
Individuals with T2D, %	2.7	5.7	8.6	10.6	14.5
T2D duration, years (SD)	5.9 (6.9)	5.0 (6.1)	4.5 (5.6)	4.3 (5.4)	4.1 (5.1)

Abbreviations: BMI, body mass index; CPRD‐HES, Clinical Practice Research Datalink‐Hospital Episode Statistics; HbA1c, glycated hemoglobin; HDL, high‐density lipoprotein; SBP, systolic blood pressure; SD, standard deviation; T2D, type 2 diabetes.

^a^ Calculated as 100% minus the proportions of individuals with pre‐T2D or T2D.

^b^ In scenario analyses that adjusted prediabetes prevalence to real‐world values, distribution at baseline was modified to 25.9% prediabetes, 2.7% T2D and 71.4% nonprediabetes and non‐T2D.

The COM was used to simulate the incidence of CV events (MI/unstable angina and stroke/TIA) and T2D, as well as all‐cause mortality. Analyses were conducted over a 10‐year time horizon in a cohort of 100 individuals and translated into event rates per 1000 patient‐years by division with the model's projected undiscounted life expectancy.

Longitudinal data reflecting changes to BMI over time were not investigated in the CPRD‐HES study; consequently, in this analysis, BMI was assumed to remain constant over time. No weight management intervention effects were considered in these analyses.

#### 2.2.2 Calculation of event rates

Cox‐adjusted event rates for each BMI group were calculated by multiplying the crude event rates in the normal weight group by the Cox‐proportional hazard ratios (HRs; Table 7) for events of interest in each BMI group. The CPRD‐HES analyses were adjusted for age, sex and smoking status. In the COM, the baseline characteristics of the reference BMI group were kept, and the mean BMI was changed per each of the simulated BMI groups according to the mean BMI reported in the CPRD‐HES study.

#### 2.2.3 Scenario analysis with altered baseline prediabetes prevalence

Laboratory test data used to derive prediabetes rates in CPRD were available for only a small proportion of the individuals in the database. Therefore, the prediabetes rates of 2.0%–6.9% across BMI groups in the CPRD‐HES study (Table 6) were considered likely to be an underestimate of rates in the general population, particularly when contrasted with the 2011 Health Survey of England (HSE), which reported rates of 25.9% in individuals with a BMI of 25 kg/m^2^ or less, 37.6% in those with a BMI of 25.0–29.9 kg/m^2^ and 47.9% in those with a BMI of 30 kg/m^2^ or above.^43^

To examine the impact of altering baseline prediabetes prevalence, a scenario analysis was conducted in which the rate of prediabetes for individuals of normal weight reported in the HSE was used for the reference group in the model. Baseline T2D prevalence was not altered in this analysis.

#### 2.2.4 Assessment of concordance

Model concordance was assessed by plotting the predicted outcomes (Y‐axis) against the observed study endpoints (X‐axis). To quantify overprediction and underprediction, an ordinary least‐squares linear regression line (OLS LRL) was fitted to the observed data, with an intercept of zero. Slope values below 1.0 suggest underprediction and values above 1.0 suggest overprediction. Coefficients of determination (*R* ^2^) were calculated for all results to quantify linear correlation between the observed and predicted outcomes in cases where OLS LRL was close to the IL.

## 3 RESULTS

### 3.1 Comparison of predictions with CPRD‐HES study data

Table 8 shows the observed and predicted values for all outcomes in the base case and scenario analyses. The incidence of CV events was slightly overpredicted by the model (8.0–9.4 per 1000 patient‐years across BMI groups) compared with observed values (7.8–8.5 across groups), as indicated by an OLS LRL slope of 1.091 (Table 9). Similar results were obtained as part of the scenario analysis where baseline prediabetes prevalence was adjusted (Table 8).

**TABLE 7:** Cox proportional hazard ratios used for Core Obesity Model predictions

	BMI	BMI	BMI	BMI	BMI
	18.5–24.9 kg/m^2^	25–29.9 kg/m^2^	30–34.9 kg/m^2^	35–39.9 kg/m^2^	40–45 kg/m^2^
Unstable angina/myocardial infarction, HR (95% CI)	1.0 (reference)	1.03 (1.02–1.04)	1.11 (1.09–1.12)	1.14 (1.12–1.17)	1.18 (1.14–1.23)
Stroke/transient ischemic attack, HR (95% CI)	1.0 (reference)	0.92 (0.91–0.94)	0.94 (0.92–0.95)	0.98 (0.95–1.00)	1.02 (0.98–1.06)
Type 2 diabetes, HR (95% CI)	1.0 (reference)	2.30 (2.27–2.34)	4.73 (4.65–4.80)	7.81 (7.67–7.96)	10.8 (10.5–11.0)
All‐cause mortality, HR (95% CI)	1.0 (reference)	0.77 (0.76–0.77)	0.81 (0.80–0.82)	0.95 (0.94–0.97)	1.21 (1.18–1.24)

Abbreviations: BMI, body mass index; CI, confidence interval; HR, hazard ratio.

**TABLE 8:** Observed event rates from the CPRD‐HES study versus those predicted by the Core Obesity Model (versions 8.0 and 6.1)

Incidence, crude event rates/1000 patient‐years	BMI	BMI	BMI	BMI	BMI
	18.5–24.9 kg/m^2^	25–29.9 kg/m^2^	30–34.9 kg/m^2^	35–39.9 kg/m^2^	40–45 kg/m^2^
**Cardiovascular events, total**					
CPRD‐HES study, observed event rates	7.8	7.6	7.9	8.2	8.5
Core Obesity Model (version 8.0), predicted					
Base case	8.0	8.3	8.7	9.2	9.4
Scenario analysis	8.3	8.6	9.1	9.7	9.9
Core Obesity Model (version 6.1), base case	6.0	6.2	6.6	7.0	7.1
**Unstable angina or myocardial infarction**					
CPRD‐HES study, observed event rates	3.4	3.5	3.8	3.9	4.0
Core Obesity Model (version 8.0), predicted					
Base case	6.0	6.3	6.6	6.9	7.1
Scenario analysis	6.2	6.5	6.9	7.3	7.5
Core Obesity Model (version 6.1), base case	4.5	4.7	4.9	5.2	5.3
**Stroke or transient ischemic attack**					
CPRD‐HES study, observed	4.4	4.1	4.1	4.3	4.5
Core Obesity Model (version 8.0), predicted					
Base case	2.0	2.1	2.2	2.3	2.3
Scenario analysis	2.0	2.1	2.3	2.4	2.5
Core Obesity Model (version 6.1), base case	1.5	1.6	1.7	1.8	1.8
**Type 2 diabetes**					
CPRD‐HES study, observed	2.1	4.8	9.9	16.4	22.7
Core Obesity Model (version 8.0), predicted					
Base case	1.0	2.2	4.2	6.6	7.6
Scenario analysis	2.7	4.8	8.0	11.6	13.1
Core Obesity Model (version 6.1), base case	1.1	2.3	4.2	6.5	7.5
**All‐cause mortality**					
CPRD‐HES study, observed	11.6	8.9	9.4	11.0	14.0
Core Obesity Model (version 8.0), predicted					
Base case	4.8	4.9	5.0	5.1	5.2
Scenario analysis	4.9	5.0	5.1	5.3	5.3
Core Obesity Model (version 6.1), base case	3.8	3.8	3.9	4.0	4.0

Abbreviations: BMI, body mass index; CPRD‐HES, Clinical Practice Research Datalink‐Hospital Episode Statistics.

**TABLE 9:** Linear regression analysis of observed event rates versus those predicted by the Core Obesity Model

Outcome	OLS LRL slope	R^2^
**Cardiovascular events (total)**		
Base case	1.091	0.750
Scenario analysis	1.141	0.719
**Type 2 diabetes**		
Base case	0.368	0.954
Scenario analysis	0.655	0.862
**All‐cause mortality**		
Base case	0.445	−26.840
Scenario analysis	0.455	−22.090

Abbreviations: OLS LRL, ordinary least‐squares linear regression line; R^2^, coefficient of determination.

Incidence of T2D was strongly linked to BMI in the CPRD‐HES study, with rates increasing exponentially from 2.1 in the normal weight group to 22.7 in those with a BMI of 40.0–45.0 kg/m^2^. A similar pattern was apparent in the model predictions; however, T2D incidence was generally underpredicted by the COM (OLS LRL slope of 0.368 [Table 9]), especially in the highest BMI group (7.6 vs. 22.7). When baseline prediabetes prevalence was increased to the level observed in the HSE survey, predicted T2D rates were consistent with observed values in the lower BMI groups (18.5–24.9 and 25.0–29.9 kg/m^2^; Table 8); however, event rates were still underpredicted in the other BMI groups (overall OLS LRL slope: 0.655; Table 9). The underprediction was highest in the 40.0–45.0 kg/m^2^ group (13.1 vs. 22.7), indicating that underestimation of prediabetes in the CPRD‐HES study contributed to the low predicted T2D rates in base case analyses.

Observed all‐cause mortality event rates showed a gradual increase across the BMI groups, with a rate of 8.9 for those with BMI between 25.0 and 29.9 kg/m^2^, rising to 14.0 for those with BMI 40.0–45.0 kg/m^2^. However, the predicted rates remained relatively constant across groups (4.9–5.2), indicating that mortality rate predictions by the COM may be insensitive to changes in BMI. This underprediction was confirmed by linear regression analysis (OLS LRL slope: 0.445; Table 9); however, the negative *R* ^2^ value obtained from this analysis (−26.840; Table 9) limited the ability to fully interpret the result. All‐cause mortality predictions were consistent in the scenario analysis (Table 8), suggesting that prediabetes prevalence did not significantly affect mortality during the modeled time horizon.

### 3.2 Comparison of predictions generated by different versions of the Core Obesity Model

As part of the development of the COM, a previous version (6.1) was subjected to an extensive validation process, according to best practice guidelines.^13^ Results from a comparison between the current (8.0) and validated (6.1) versions of the COM (Table 8) showed that the validated version produced similar trends across BMI groups to those predicted by the current version, but that predicted event rates were generally slightly lower.

## 4 DISCUSSION

Economic models can be used to extrapolate the long‐term impacts of a disease and estimate the relative benefits of different treatment strategies. In conjunction with shorter‐term data provided by clinical trials and observational studies, such projections are relevant to clinicians, payers, and policy‐makers, particularly in the case of a common, chronic condition such as obesity. Best‐practice guidance highlights the need for transparency and validation to ensure that the outputs of economic models can be interpreted with confidence by all stakeholders.^13^ The aim of this study was to present the structure and components of the COM in a transparent manner and provide a single‐study example of its predictive ability.

The COM incorporates a broad range of obesity‐related health states, allowing for the presence of single and multiple comorbidities, and including complications both strongly and moderately related to obesity. When deriving data to develop risk equations for these complications, multiple relevant studies were considered, and those that were most appropriate based on study population and setting were selected. In their final appraisal determination for liraglutide, NICE judged that the model health states and transitions are suitable for decision‐making.^44^ The COM also improves on previously developed models via the inclusion of a greater range of obesity‐related complications, as well as incorporating the effects of baseline prediabetes. The comparative analysis, in which adjustment of prediabetes at baseline strongly influenced the prediction of T2D in the model, demonstrates the importance of including this factor in models of obesity. The relevance of prediabetes as a baseline factor is supported by data indicating that individuals with this condition have a 33%–66% risk of developing T2D within 3–6 years, as well as an elevated risk of CVD compared with the general population.^45^

Predicted rates of CV events in the COM were well matched with those observed in the CPRD‐HES study in both base‐case and scenario analyses; however, overprediction of unstable angina/MI rates and underprediction of stroke/TIA rates highlighted the role that repartitioning can play during predictions of composite endpoints. All‐cause mortality event rate predictions were lower than observed, which demonstrates the continuing refinement required for economic models, including the potential for the addition of further obesity‐related complications, such as chronic kidney disease, as well as highlighting the incomplete understanding of the relationship between BMI and mortality. In the CPRD‐HES study, as in several previous analyses,^46^, ^47^, ^48^, ^49^, ^50^ mortality was higher in individuals with a BMI of 18.5–24.9 kg/m^2^ than in those with a BMI of 25.0–29.9 or 30.0–34.9 kg/m^2^. This may be partly attributable to unintentional, pre‐diagnostic weight loss in individuals at high risk of death, meaning that they are represented disproportionately in the lowest BMI group; however, further research is required to understand the contribution of BMI and other demographic and disease risk factors to mortality. Notably, all patients entered the COM free of CVD; however, this was not the case for patients in the CPRD‐HES study.

It must also be noted that mortality rates in the CPRD‐HES study (index period: January 2000–December 2010) are higher than those reported in several more recent studies. The 11.6%–14.0% mortality across BMI groups in this data set contrasts with rates of 7.1% in a study conducted by the Global BMI Mortality Collaboration,^48^ 8.0% in a 2018 study using CPRD data,^47^ and 3.9%^51^ and 4.0%,^50^ respectively, in studies published in 2019 using data from the UK Biobank. This pattern is supported by the findings of a study that examined mortality in five survey periods from 1986 to 2009, which concluded that mortality is decreasing over time.^52^ Such trends may be attributable, in part, to improvements in the management of obesity‐related diseases during more recent decades. Therefore, the fact that mortality estimates generated by the COM are low compared with the rates observed in the CPRD‐HES data set may be partly explained by the lower general population mortality informing the non‐disease‐specific mortality in the COM (based on 2019 England and Wales general population mortality statistics published by the Office of National Statistics), in line with the observed trend in decreasing population mortality over the past decade.

The results of this single‐study comparison should also be considered in the context of the published COM external validation publication,^41^ which provides a more robust analysis of prediction accuracy against a larger number of studies. It should also be noted that when comparing the cost‐effectiveness of weight management interventions, any misprediction of mortality or other factors applies equally to both treatments being assessed, minimizing the risk of bias. Furthermore, the ISPOR/SMDM guidelines do not quantify the desired level of accuracy for the predictions made by models and emphasize that such quantification would not be feasible or useful, stating that “it is not possible to specify criteria that a model must meet to be declared ‘valid’, as if validity were a property of the model that applies to all of its applications and uses for all time.”^12^ ^,^ ^13^

Taken together, the comparisons of observed and predicted values performed here provide further insight into the results of the previously reported external validation^41^ and highlight the importance of baseline prediabetes prevalence. This analysis also indicates areas for further improvement and refinement in the COM. Adjustments to the COM are ongoing, in line with identification of new evidence; however, the present study provides an example of the model's functionality at this point in time, based on currently available published studies. Furthermore, the trends in the predictions generated by the present version of the COM (well‐matched predictions for CV events and for T2D in the normal and overweight BMI groups and underprediction of mortality across BMI groups) were consistent with a previous, extensively validated, version of the COM.^41^

Predictive models and the economic analyses performed by them are necessarily limited by the quality and scope of the data available. For example, in the COM, some of the studies used to derive risk estimates did not include BMI as an independent risk factor^28^ or did not estimate the impact of BMI above a certain threshold.^22^ ^,^ ^23^ ^,^ ^26^ Therefore, the COM may underpredict disease risk for individuals with a BMI greater than 40 kg/m^2^; this is reflected in the predicted T2D incidence in these analyses, which was lower than observed values in the highest BMI group. Furthermore, the COM is intended to reflect clinical practice as accurately as possible; however, epidemiological and database studies cannot capture all factors that affect obesity and disease risk. Adherence to and persistence with medication, as well as demographic characteristics and medical history, which may constitute important risk factors, are unlikely to be recorded fully in these databases. For example, socioeconomic status is implicated in a considerable proportion of obesity^53^ but is not captured in CPRD or similar retrospective data sources. Finally, outcomes relating to CVD risk equations were subject to some assumptions as a result of the source material available: the risk of CVD as a first‐time event was assumed to be the same for individuals with normal glucose tolerance and for those with prediabetes, and once an individual developed prediabetes, their risk of CVD as a recurrent event was the same as for those with T2D.

The COM improves on previous economic models of obesity^8^, ^9^, ^10^, ^11^ due to the inclusion of additional health states and baseline characteristics. The results of this study show that in the context of the UK clinical practice, the COM can predict rates of CV events across BMI groups and T2D in certain BMI groups, both of which are strongly linked to obesity. Further adjustment to the model prediction of mortality rates, especially at higher BMI levels, will improve and refine its overall ability to estimate the occurrence and health economic burden of obesity‐related complications, providing a valuable tool to support healthcare decision‐making.

## CONFLICT OF INTEREST

Sandra Lopes and Henrik H. Meincke are employees and shareholders of Novo Nordisk A/S. IQVIA, the employer of Mark Lamotte and Anamaria‐Vera Olivieri, received consulting fees from Novo Nordisk A/S for this study. Michael E.J. Lean has received departmental funding and contributed to advisory boards for Novo Nordisk.

## AUTHOR CONTRIBUTIONS

All authors contributed to the study design, data interpretation, and writing and critical review of manuscript content. Sandra Lopes, Mark Lamotte, and Anamaria‐Vera Olivieri were involved in performing and reviewing the data analysis.
